# Implementation of a Clinical Pharmacy Education Program in a Teaching Hospital: Resident Oriented Documentation and Intervention

**Published:** 2010

**Authors:** Fanak Fahimi

**Affiliations:** a *School of Pharmacy, Shahid Beheshti, University of Medical Sciences, Tehran, Iran.*; b * Pharmaceutical Care Department, Chronic Respiratory Disease Research Center, TB and Lung Disease Research Center, NRITLD, Masih Daneshvari Hospital, Shahid Beheshti University of Medical Sciences, Tehran, Iran.*

**Keywords:** Education, Clinical Pharmacy, Intervention, Interventions, Medication

## Abstract

The aim of this study was to provide a clinical pharmacy education program at Masih Daneshvari hospital, a University affiliated hospital, located in Tehran, Iran.

For this purpose, the most common pharmacist involved interventions and aspects of potential fields for pharmacy students and residents education was firstly identified. Clinical pharmacy interventions and drug information forms were filled during the study period, from January 2006 till January 2007.

Based on the results of this study, a total number of 772 interventions were conducted during the study year. Drug information had the highest rate of 22.30% among all interventions, followed by dose adjustment, and therapeutic reduction or addition. The mean number of medications per patient was 8.62 ± 7.54.

In conclusion, it could be said that although in our country the challenge for the pharmacy as a profession is in its initial stages compared to the developed countries, the result of this study revealed a high demand for this service among health care providers.

## Introduction

Clinical pharmacists started their contributions to medicine in the 1960s and have come to be crucial members of medical teams as seen today in hospitals in developed countries. The presence of clinical pharmacists resulted in a safer medication administration, better patient outcomes, lower use and therefore lower costs of drugs. In addition, their presence has led to a higher quality of patient education and provision of complete detailed information for patients (1).

Documentation is the milestone to display the value of interventions within an organized health care system. Pharmaceutical care is not an exception to this rule (2). A major element of the idea of pharmaceutical care is the documentation of provided care (3). 

Documentation enables the pharmaceutical care model of pharmacy practice to make the best communication with the third-party system of reimbursement payment (i.e. insurance companies). Communication among sites of patient care must be precise and appropriate to make pharmaceutical care possible. The lack of universal reimbursement for services provided by pharmacist can serve a road block for initiating documentation (4). However, the chance to demonstrate contributions to patient outcomes and safety must serve as a catalyst for pharmacist to document their services (2). 

One study conducted on a general medicine unit showed that pharmacist involvement with medical doctors could be associated with a considerable reduction in preventable Adverse Drug Events (ADEs) (5). Another study demonstrated a positive contribution of pharmacists` interventions and quality of pharmacotherapy in 141 Dutch community pharmacies (6). Dooley and colleagues conducted a prospective study and examined the pharmacists’ interventions in eight hospitals in Australia. The results showed a considerable pharmacist-related cost savings of $4,444,794 (7). Results of a research at an outpatient study showed that regardless of the setting, pharmacists could improve patient outcomes (8). Van Wijk and colleagues reviewed 18 trials and failed to show a significant patient adherence to chronic medical therapies (9) American College of Clinical Pharmacy (ACCP) noted a decrease in cost by clinical pharmacy services in most studies which were done from 1996 to 2000 (10). Using published relevant articles between 2001 and 2005, ACCP has also showed continued benefits of these services on cost reduction (11). In Iran, the professional movement of pharmacists and their efficient involvement in hospital wards at Shahid Beheshti University, Tehran, has begun in recent years and needs more momentum (12). An opportunity to take up this challenge was provided in 2005. We initiated clinical pharmacy services to interested bodies of Masih Daneshvari Hospital. The precise nature of this service was vague at first, but in subsequent discussions it was well defined. 

The Adverse Drug Reaction (ADR) unit at Masih Daneshvari Hospital was formally established in 2006 (13) and has a clinical pharmacist as the chief of the unit and preceptor. The ADR unit focuses on the following activities: ensuring safe and cost effective drug administration, monitoring and management of drug use patterns, providing drug information, training clinical pharmacy residents and pharmacy students, publishing monthly ADR bulletins, running anticoagulant clinic, and providing drug protocols and treatment guidelines. 

The clinical pharmacist intervenes with medical therapies by either random visits of the patients, continuous monitoring of drug utilization, and providing education to health care providers. Clinical pharmacy services at the hospital are aiming to start various interventions in different wards to reduce the adverse drug effects, to eliminate unnecessary drug administrations and to lower the costs of therapy. In the current study, we have reported the results of one year experience of clinical pharmacy establishment program at this hospital. 

## Experimental

This study was conducted in a 300-bed respiratory hospital between January 2006 to January 2007. The clinical pharmacy program was designed to provide an opportunity for pharmacy students to attain education, while offering clinical services to patients at Masih Daneshvari hospital. Clinical pharmacy residents spend two months rotation in pulmonology ward as part of their residency program (RP). The last 2 years of RP consists of eighteen months of different hospital ward rotations. Residents are expected to fill in clinical pharmacy intervention and drug information forms during their educational program. In the first month of the rotation, an intensive training program was provided to the residents introducing pharmaceutical care philosophy and information gathering. In the second month the residents visited the patients independently and/or intervened during the clinical rounds. This is also consistent with the current Doctor of Pharmacy program, during which students will rotate at a specific site for a limited period of time. 

All medication regimens were recorded and patient medication profiles were generated, and updated on each subsequent visit. Each patient was visited by pharmacy students/residents on the basis of receiving written consultation request from the physician in charge or as a verbal request during the clinical rounds. Medication counseling/advice was also given to the patient upon physician, nurse or patient request. In addition, patients were instructed on their medication use e.g. inhaler technique.

All consultation requests were answered back by the preceptor, or verified if the recommendations had been written by the residents. All the forms were signed by the preceptor thereafter. The residents and their preceptor discussed and reviewed the patient medication histories and extracted Drug Related Problems (DRPs). Patients` charts were reviewed to identify DRPs. Relevant labaratory data (e.g. platelet count, and serum creatinin) and vital signs (e.g. blood pressure and pulse rate) were all recorded to support the appropriateness of the interventions. 

Supporting literature was provided to each resident for the recommendations made. For the DRPs, a recommendation was formed after group discussion. A pharmacotherapy sheet or the consultation form was then put in the patients` chart for the physician. Interventions were categorized into 11 categories, which are defined as follows:

*Dose adjustment: *adjusting doses for patients with renal or hepatic impairment, elderly patients, or those receiving inappropriate doses according to the indication.

*Therapeutic reduction or addition: *changing drug dosage regimens following side effects or para-clinical tests. 

*Order clarification/ patient education/ compliance: *providing education to patients in order to increase compliance or the clarification of the order. This was more conducted at the time of discharge for patients who needed to continue therapy. 

*Monitoring recommendations: *recommending monitoring to avoid or explore toxicity and a possible side effect or efficacy (e.g. platelet monitoring for heparin induced thrombocytopenia, INR for warfarin). 

*Drug interaction: *counseling on pharmacody- namic and pharmacokinetic interactions that may arise when two or more drugs are used at the same time. 

*Therapy interchange/ changing routes of administration: *recommending alternative drugs with similar indication for a special patient (e.g. enoxaparin versus heparin, IV to PO).

*Staff error/ transcription error correction: *correcting errors that has been made by the staff or transcription of the order from the previous step.

*Therapy duplicate: *eliminating redundant drug therapies to improve patient safety.

*Pre-op: *counseling on the medications before an elective operation.

*Allergy alert: *alerting nurses or physicians on a potential allergic reaction to drugs. 

*Other drug information (DI): *providing any information on medications on request of a health care provider.

Patient-medication exposures per day were calculated as follows: [(number of patients) x (mean number of prescribed medications)]. 

Percentage of interventions per patient-medication exposure was calculated as follows: 100 x [(number of interventions recorded) / (patient medication exposures)]. The rate of interventions per day was calculated as follows: [(total number of interventions) x (intervention recording duration (day))] (14).

## Results

**Figure 1:**
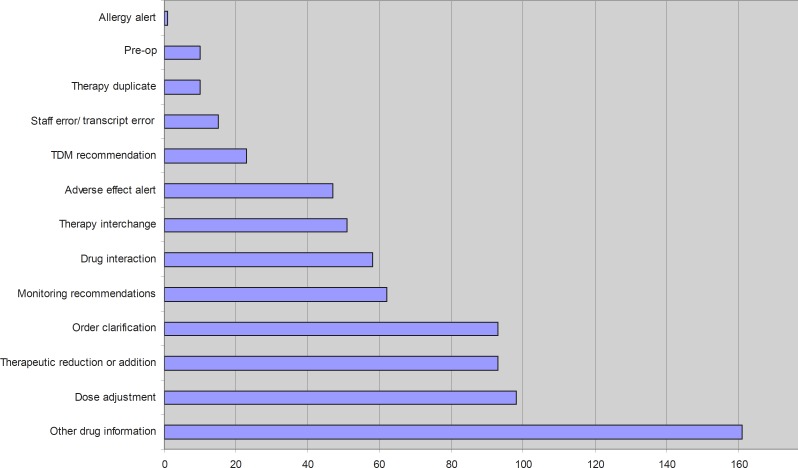
Diagram of the number of interventions in each related category

A total number of 772 interventions for 345 patients have been performed during the study year, which gives us an average of 2.70 interventions per day excluding weekend (54 days) and holidays (25 days). Drug information had the highest rate (22.30%) of all interventions, followed by dose adjustment, therapeutic reduction/ addition and patient education with 13.57%, 12.88%, and 12.88% of the interventions, respectively (Table 1 and Figure 1).

**Table 1:** Number and percentage of interventions in related categories

**Category **	**N **	**Percentage **
Other drug information	161	22.30
Dose adjustment	98	13.57
Therapeutic reduction or addition	93	12.88
Order clarification/ patient education/ compliance	93	12.88
Monitoring recommendations	62	8.59
Drug interaction	58	8.03
Therapy interchange/ changing routes of administration (i.e. IV to PO…)	51	7.06
Adverse effect alert	47	6.51
TDM recommendation	23	3.19
Staff error/ transcript error	15	2.08
Therapy duplicate	10	1.39
Pre-op	10	1.39
Allergy alert	1	0.14
**Total **	**722 **	**100.00 **

Patient-medication exposures per day were 2974 cases. Each patient was taking 8.62 ± 7.54 (mean ± SD) medications per hospital stay. Percentage of interventions per patient-medication exposure was calculated as 100 x (722 / 2974) = 24.28%.

## Discussion

The number of interventions recorded in this study was 722 cases. This includes only patients admitted to the hospital. This is very similar to a study performed by Japanese researchers who reported a total of 347 interventions for 164 patients during a 6 month period (15). 

Based on an one Iranian study published recently, only six percent of a total number of 188 patients who were consulted on the interval and dosing of their medications received proper information (16). It is obvious that there is a considerable need for improving pharmacists` services in both community and hospital setting. 

The high rate of drug information (DI) requests (22.30% of all interventions) justifies the fact that clinical pharmacy program should fill the gap between practice and pharmaceutical information. Information on the new drugs was mainly received from pharmaceutical company representatives, (17). which could sometimes be biased and misleading in clinical practice. 

The mean number of drug administration for each patient was reported as 8.62 in this study. 

It has been shown that prescription of four medications or more can put the patient at a great risk of drug related morbidity (18). The original aim of clinical pharmacy as a professional practice (and not a health science) is providing pharmaceutical care (19). Education of pharmacy students and clinical pharmacy residents, while providing valuable care component to patients and other health-care providers, can successfully be performed as shown by the results of our program implemented in a teaching hospital. We understand that this program is a novel approach to the education of pharmacy students and clinical pharmacy residents in our country. Though, no data is available on the quality of student learning by this method of education. Also, we have no data on nursing and physicians perceptions of the program. As this program grows, we hope to find answers to these questions. 

The knowledge gained at this stage and desired outcomes can be applied in the education of pharmacy and medical students. This implemented program of education has mostly met the desired goals, as it is obvious in the case of the number of the interventions. High acceptance rate was reported by other authors too (14). 

Shahid Beheshti School of Pharmacy persists that the challenge for the pharmacy as a profession is to switch from theoretical education to practical bedside management of patients` medication. Achieving this goal is equal to the development of clinical pharmacy programs in the teaching hospitals. It is believed that this plays a major role in the future of pharmacy.

Here, we did not report the results of improvement in the quality of pharmaceutical care or the lectures which were delivered in this regard in the hospital. Also, the anticoagulation clinic which is run by pharmacists as part of pharmaceutical care department was not discussed in this study. 

There are limitations in our study, which should be acknowledged in interpretation of the obtained results. Lack of cooperation between patients and doctors in our report may lead to some biases. Culturally, Iranian patients do not easily report detailed information on drugs taken and that may result in non-comprehensive results. We could only rely on charts and patient records as source of drug therapy, though drug history taking was also tried. Many surgeons are not willing to consult the necessary pre-op drug adjustment or post-op drug intake with pharmacists. Since this program is quite new in Iran, it has not yet been completely presented to either patients or medical doctors. 
